# Isolation-Induced Ultrasonic Vocalization in Environmental and Genetic Mice Models of Autism

**DOI:** 10.3389/fnins.2021.769670

**Published:** 2021-11-22

**Authors:** Itay Shekel, Shaked Giladi, Eynav Raykin, May Weiner, Vered Chalifa-Caspi, Dror Lederman, Ora Kofman, Hava M. Golan

**Affiliations:** ^1^Department of Physiology and Cell Biology, Ben-Gurion University of the Negev, Be’er Sheva, Israel; ^2^Zlotowski Center for Neuroscience, Ben-Gurion University of the Negev, Be’er Sheva, Israel; ^3^Department of Psychology, Ben-Gurion University of the Negev, Be’er Sheva, Israel; ^4^Ilse Katz Institute for Nanoscale Science and Technology, Ben-Gurion University of the Negev, Be’er Sheva, Israel; ^5^Faculty of Engineering, Holon Institute of Technology, Holon, Israel; ^6^National Center for Autism Research, Ben-Gurion University of the Negev, Be’er Sheva, Israel

**Keywords:** autism, MTHFR, chlorpyrifos, GABA, ultrasonic vocalization, communication, newborn

## Abstract

Studies in rodent models suggest that calls emitted by isolated pups serve as an early behavioral manifestation of communication deficits and autistic like behavior. Previous studies in our labs showed that gestational exposure to the pesticide chlorpyrifos (CPF) and the Mthfr-knock-out mice are associated with impaired social preference and restricted or repetitive behavior. To extend these studies, we examine how pup communication *via* ultrasonic vocalizations is altered in these ASD models. We implemented an unsupervised hierarchical clustering method based on the spectral properties of the syllables in order to exploit syllable classification to homogeneous categories while avoiding over-categorization. Comparative exploration of the spectral and temporal aspects of syllables emitted by pups in two ASD models point to the following: (1) Most clusters showed a significant effect of the ASD factor on the start and end frequencies and bandwidth and (2) The highest percent change due to the ASD factor was on the bandwidth and duration. In addition, we found sex differences in the spectral and temporal properties of the calls in both control groups as well as an interaction between sex and the gene/environment factor. Considering the basal differences in the characteristics of syllables emitted by pups of the C57Bl/6 and Balb/c strains used as a background in the two models, we suggest that the above spectral-temporal parameters start frequency, bandwidth, and duration are the most sensitive USV features that may represent developmental changes in ASD models.

## Introduction

Autism spectrum disorder (ASD) refers to a multi-faceted developmental syndrome whose symptoms include impoverished verbal and non-verbal communication, atypical social interactions, and repetitive behaviors. As in other complex behavioral disorders, genetic and pharmacological mouse models are used to investigate the genetic and neurological substrates of the syndrome. Recent research indicates that signs of ASD can be detected in babies even before the emergence of verbal speech. Retrospective analysis of canonical and non-canonical vocalization in infants, who were later diagnosed with ASD, showed lower production of vocalizations compared to typically developing babies (Patten et al., 2014). Siblings of children diagnosed with ASD, considered high risk for ASD, produced fewer canonical syllables, consonant types and speech-like vocalizations, and more non-speech vocalizations than children at low risk for ASD (Paul et al., 2011). Pre-speech vocalizations in infants are modified by interaction with the caregiver, causing perturbation of social dynamics in babies who were later diagnosed with ASD. For example, adults reacted more to non-speech-like vocalizations for the babies who were later diagnosed with ASD because their canonical babbling was impoverished (Warlaumont et al., 2014).

Ultrasonic vocalizations (USV) production in isolated mouse pups provides a potential assay for assessing communication deficits in mouse models of ASD. Pup calls enhance maternal approach and retrieval behaviors (Ehret, 2005). The maternal response suggests that USVs have adaptive communicative functions. In addition, responsivity of the dam has established USVs as “*an important indication of responsivity to social stimuli*” (Kazdoba et al., 2016) reinforced by post-isolation reunion with the dam and litter (Hofer and Shair, 1991).

Epidemiological evidence favors a genetic etiology for ASD (Satterstrom et al., 2020); however, incomplete concordance between monozygotic twins suggests that there is also an environmental component (Hallmayer et al., 2011). Longitudinal studies on toddlers who were exposed to pesticides *in utero* in agricultural and urban communities in the US and China pointed to an association between levels of organophosphate (OP) pesticide metabolites and impaired social development in the toddlers (Rauh et al., 2006; Eskenazi et al., 2007; Wang et al., 2017). Impaired social skills in black participants and in boys aged 7–9 years were associated with gestational exposure to OP pesticides (Furlong et al., 2014; Shelton et al., 2014). These studies point to a possible contribution of gestational OP pesticide exposure to ASD symptoms.

The Methylenetetrahydrofolate reductase (Mthfr) gene (Entrez Gene ID: 4524) encodes for an essential enzyme in the C1 metabolic pathway. The Mthfr 677C > T polymorphism (rs1801133), which shows suppressed activity of this enzyme, is markedly higher among ASD patients and their mothers than in the general population (Goin-Kochel et al., 2009; Mohammad et al., 2009; Schmidt et al., 2011); see also meta-analysis by Rai (2016).

Previous work in our labs showed that gestational exposure to the pesticide chlorpyrifos (C_9_H_11_C_l3_NO_3_PS, CID2730, CPF) (Lan et al., 2017, 2019) and the Mthfr heterozygosity (Mthfr+/−) in mice (Sadigurschi and Golan, 2018; Orenbuch et al., 2019; Agam et al., 2020) are both associated with impaired social preference and restricted or repetitive behavior, establishing them as mouse models of ASD with face validity. Mouse pups in both models present with developmental delay, in agreement with a neurodevelopmental delay observed in part of ASD patients (Furlong et al., 2014; Shelton et al., 2014).

Gestational administration of the pesticide CPF (1 mg/kg) in the second-third trimester to mouse dams induced a reduction in the number of USV calls and longer latencies to produce calls, with no difference in call duration or the highest or mean peak frequency in pups (Morales-Navas et al., 2020). A similar regimen of *in utero* CPF (6 mg/kg) tested in BTBR mice and CPF-oxon in C57Bl6 mice did not alter the number of isolation-induced USV calls compared to vehicle treated pups (Laviola et al., 2006; De Felice et al., 2015), but did reduce the number of USVs in CD1 outbred pups (Venerosi et al., 2009).

Studies that analyzed USV in ASD mouse models suggest that quantitative changes in USV production that were described in various ASD genetic models are variable. For example, the Engrailed-2 null (Brielmaier et al., 2012), the Avpr1b (Scattoni et al., 2008a), and the FMR1 knock-out (KO) pups (Nolan et al., 2017, 2020) did not produce significantly fewer isolation calls than the control wild-type mice. On the other hand, other genotypes such as BTBR (Scattoni et al., 2008b) and FMR1-KO on postnatal day 7 (P7) (Lai et al., 2014) that clearly showed impaired social behavior show greater production of USV than the control genotype. Thus, despite deficits in social and repetitive behavior in the different mouse models, the quantity of USVs can be greater than, less than, or not different from the USV production in the control genotype (Lai et al., 2014). Considering the variability of phenotypes in ASD, and mismatch between the severity of communication impairment and rigid behaviors, it is not surprising to find vast variability when considering only the number of calls produced. Call duration has also been suggested as a valid variable to distinguish between ASD-model mice and wild-type controls as demonstrated by the BTBR strain, FMR1-KO (Lai et al., 2014; Nolan et al., 2020), and females of the ProSap1/Shank 2 (Ey et al., 2013) genetic models of ASD.

Considering the variable phenotypes in ASD models and the emerging finding in babies’ spectral properties, the major goal was to describe common and diverse USV features of a genetic and an environmental ASD mouse model using a spectral-temporal analysis of the characteristics of the USVs in order to compare the two models. To do this, we used a novel, unsupervised spectral analysis of the call syllables which allowed us to define strain and sex differences as well as the ASD-relevant features that have not been described previously.

## Materials and Methods

### Experimental Design

#### Methylenetetrahydrofolate Reductase Mice

Mice on a Balb/cAnNCrlBR background were studied (Chen et al., 2001) to assess the effect of maternal Mthfr+/− genotype vs. offspring genotype. Mthfr+/+ [wild-type (Wt)] and Mthfr+/− [heterozygote (Het)] female mice were mated with Wt males to create three groups defined by genotype and maternal genotype as follows: Wt offspring from Wt mothers (Wt:Wt, *n* = 23), Wt offspring from Mthfr+/− mothers (Het:Wt, *n* = 18), and Mthfr+/− offspring from Mthfr+/− mothers (Het:Het, *n* = 21). Mthfr−/− mice are not viable.

#### C57Bl6J (B6) Mice

Dams and sires for breeding were purchased from Envigo, Israel. Dams were divided into three treatment groups for pesticide exposure (CPF) during gestation: Vehicle (VEH, corn oil, *n* = 17), 2.5 mg/kg CPF (CPF-L, *n* = 22), or 5 mg/kg CPF (CPF-H, *n* = 20).

Sample size was chosen based on our previous experiments. An illustration of the study design and description of experimental groups can be seen in Supplementary Figure 1A.

The mouse colonies were maintained on a 12:12 h light/dark schedule, temperature 21–23°C with *ad libitum* food and water. All procedures were performed according to the guidelines of the Israeli Council on Animal Care and approved by the Animal Care and Use Committee of Ben-Gurion University of the Negev (protocols IL-16-07-14 and IL-66-11-13).

### Prenatal Exposure

Chlorpyrifos (CPF, 99.5% purity, Chem Service, Inc) suspended in corn oil (Willi Food, Yavneh, Israel) or vehicle control was administered by gavage to B6 pregnant females daily from gestation day (GND) 12–15 in a volume of 0.1 ml/10 g body weight using a 22-gauge stainless steel feeding tube (Solomon Instech, Inc). The dams showed no signs of cholinergic toxicity (Ricceri et al., 2007; Lan et al., 2017).

### Genotyping

Mthfr and Wt mice were genotyped using polymerase chain reaction, as previously described (Chen et al., 2001).

### Ultrasonic Vocalization

#### Acoustic Recording

Ultrasonic signals were recorded using Avisoft Bioacoustics (Berlin, Germany) system including UltraSoundGate 116Hm with the Ultrasound Microphone CM16/CMPA, using the Avisoft Recorder 4.2.17 – Bioacoustics recording software. Recordings were set at a sampling frequency of 250 kHz in a trigger mode using a threshold of 0.5% of the signal’s energy in the range of 10–250 kHz.

#### ASD Models

Methylenetetrahydrofolate reductase model: two to three pups per litter were recorded on P4, 6, 8, 10, and 12. Sex and genotype were defined when pups reached P30. CPF model: One male and one female pup from each litter, marked by snipping the end of the tails on P1, were randomly chosen. Pups were recorded on P2, 5, 8, and 14. To enable comparison between the environmental and genetic model, the third recording sessions that took place at P8 were analyzed for the current study, as in other studies on analysis of pup USV communication (De Felice et al., 2015). Data collected on other postnatal days will be used for further developmental studies. In total, the numbers of mice analyzed for the current study were as follows: Mthfr, 31 each sex; CPF, 31 female and 28 male.

Each pup was separated from the litter and placed in a transparent plastic cup (11 cm high and 10 cm diameter). The microphone was placed 10 cm above the pup. After a 10-min isolation session, the pup was placed back in the home cage with the litter and the area cleaned with ethanol (70%) between pups.

#### Ultrasonic Vocalization Analysis

Call variables were extracted by SASLab Pro (Avisoft Bioacoustics^®^, Berlin, Germany) by researchers blind to the group identity with the following setting: Single threshold of −73 dB, 5 ms hold time, and eight regular intervals of duration resulting in nine frequency samples for each syllable F1–F9 (see an example in Supplementary Figure 1B). Larger number of frequency samples (up to 30) for USV detection did not increase detection rate. The following variables were used to compare USV syllables between groups: Start Frequency, defined as mean frequency at start of the syllable - F1; End Frequency, defined as mean frequency at the end of the syllable – F9; Mean Frequency (mean of all nine samples); Bandwidth (the range of syllable’s frequencies calculated by the highest frequency sample minus the lowest frequency sample of the syllable); and Duration (the time of F9–F1).

#### Cluster Analysis

Hierarchical clustering was applied using frequency values of F1-F9 as the features for clustering (Partek^®^ software). Euclidean distance, as point distance metric, and Wards linkage as cluster distance metric were applied. The difference in the characteristics between neighboring clusters in each strain was calculated by SPSS (Kolmogorov-Smirnov, K-S test) for the five variables: start frequency, end frequency, mean frequency, bandwidth, and duration. A significant difference in at least four out of five variables was sufficient to accept the cluster as a distinct cluster. In both strains, eight distinct clusters were identified (inclusion of other parameters such as duration, bandwidth, and mean frequency did not contribute and require modification of data in order to use a single scale).

### Statistical Analysis

All calls emitted by the study pups during 10 min maternal isolation session were analyzed: 5837 syllables from the Mthfr model and 10715 from the CPF model. Statistical analyses were performed by SPSS23 software (IBM) to test the effect of the fixed factors: strain, sex, and experimental group. Analysis of parametric variables was performed by univariate (general linear model) test and test of normality. Non-parametric analysis was performed using the Kolmogorov-Smirnov (K-S) test. Syllable distribution among clusters was compared using Chi-square test.

## Results

Different mouse strains present significant variability in USV properties including number of calls, syllable duration, and distinct developmental time course (Scattoni et al., 2008b), in parallel with strain differences observed in other aspects of social behavior (Nadler et al., 2004; Panksepp et al., 2007; Defensor et al., 2011). Therefore, prior to the comprehensive analysis of environmental and genetic mouse models of autism, which are based on different strains of mice, we compared the two control groups (Wt:Wt for the Mhtfr model and oil gavage for the CPF model) for each of the models, including both sexes.

To explore USV syllables properties, five variables for each syllable were tested for normality using ShapiroWilk test; start, end, mean frequencies, bandwidth, and duration all differ from normal distribution (The distributions of these variables in the control group of the ASD models tested B6 and Balb/c strains are presented in Supplementary Figure 2).

A significant sex difference was observed in both strains for each of the variables, as shown in Supplementary Table 1. B6 and Balb/c strains differ in all variables tested: start, end and mean frequencies, duration, and bandwidth. Longer syllable duration and wider bandwidth were observed in mice of the Balb/c strain, compared to the B6 with a median value in Balb/c mice twice that of the B6. Significant differences between strains were observed also in the start and end frequencies as well as the mean frequency of syllables.

### The Effect of Chlorpyrifos and Methylenetetrahydrofolate Reductase on Ultrasonic Vocalization Spectral and Temporal Characteristics

The CPF and Mthfr models were analyzed compared to their respective control groups with sex and treatment/genotype as independent variables. An effect of *in utero* exposure to CPF at high and low doses was observed, with an interaction between sex and treatment for all variables (Table 1). In females, the effect of the low dose was significant for the start and end frequencies, bandwidth, and duration (Table 1A), and the high CPF dose increased bandwidth, end frequency, and duration as shown in Table 1A. In the males, both doses of CPF were associated with a lower start frequency and the low dose was also associated with lower mean and end frequencies (Table 1).

**TABLE 1:** The effect of CPF treatment and sex on isolation induced syllables.

**Group**		**Start (Hz)**	**End (Hz)**	**MF (Hz)**	**BW (Hz)**	**Dur (s)**
**Female**						
Vehicle	Mean	76356	76400	76761	19091	0.042
*n* = 1380	Median	72000	78255	75378	14251	0.039
	*SD*	14663	14305	12605	14237	0.034
CPF-L	Mean	74034	74463	73885	16755	0.039
*n* = 2819	Median	68590	75158	70842	10827	0.033
	*SD*	16131	16015	14220	14500	0.026
CPF-H	Mean	75837	78867	76139	21055	0.045
*n* = 2614	Median	70800	82359	73944	15876	0.040
	*SD*	14899	15507	12687	15512	0.037
**Male**						
Vehicle	Mean	79143	77264	78410	17751	0.032
*n* = 757	Median	76154	78100	77644	11985	0.030
	*SD*	16427	14571	14156	15007	0.020
CPF-L	Mean	75430	75225	75474	16280	0.037
*n* = 1925	Median	68754	76018	72513	10465	0.034
	*SD*	16929	15312	14670	14566	0.023
CPF-H	Mean	74832	77004	75006	18829	0.039
*n* = 1221	Median	68004	76400	72978	13221	0.035
	*SD*	14926	13991	12304	15270	0.024
Treatment	*F*	25.738	41.303	31.614	51.945	27.742
df 2, 10710	Sig.	**0.000**	**0.000**	**0.000**	**0.000**	**0.000**
Sex	*F*	9.962	0.059	5.881	17.948	89.964
df 1, 10710	Sig.	**0.002**	0.809	**0.015**	**0.000**	**0.000**
Treat * sex	*F*	10.230	8.392	11.305	3.361	10.320
df 2, 10710	Sig.	**0.000**	**0.000**	**0.000**	**0.035**	**0.000**

**(A)**							

**Sex**		**Treat**	**Start**	**End**	**MF**	**BW**	**Dur**

F	Vehicle	CPF-L	0.000	0.000	1.000	0.000	0.020
		CPF-H	0.930	0.000	0.519	0.000	0.000
M	Vehicle	CPF-L	0.000	0.004	0.011	0.064	0.084
		CPF-H	0.000	1.000	0.345	0.352	0.347

*Descriptive values and statistics. CPF-L, CPF low dose; CPF-H, CPF high dose; MF, mean frequency; BW, bandwidth; Dur, duration. Main effects (two- and three-way ANOVA) are presented for each model, in the lower part of each table. N, number of calls. Table 1A summarizes the results of post-hoc tests.*

In the Mthfr model, offspring and maternal genotypes interacted to affect all variables, and each of these factors interacted with sex to affect the end frequency, mean frequency, and bandwidth. Duration was affected by a maternal genotype x sex interaction (see statistical values in Table 2).

**TABLE 2:** The effect of Mthfr genotype and sex on isolation induced syllables.

**Group**	**Variable**	**Start (Hz)**	**End (Hz)**	**MF (Hz)**	**BW (Hz)**	**Dur (s)**
**Female**						
Wt-Wt	Mean	76238	68631	68011	28706	0.072
*n* = 652	Median	79076	70014	66100	30200	0.077
	*SD*	12384	14120	9260	14794	0.023
Het-Wt	Mean	74485	70095	68316	25635	0.063
*n* = 1929	Median	76441	71079	65749	26526	0.066
	*SD*	10736	14311	10052	12937	0.023
Het-Het	Mean	71888	67283	66362	24438	0.067
*n* = 581	Median	73235	69585	63958	23792	0.071
	*SD*	10767	12578	8801	12363	0.022
**Male**						
Wt-Wt	Mean	70621	67916	66515	23918	0.064
*n* = 837	Median	71770	69862	64453	24814	0.069
	*SD*	12211	13074	8630	14483	0.025
Het-Wt	Mean	68953	66576	64135	24460	0.063
*n* = 540	Median	69581	67604	61765	23921	0.065
	*SD*	16409	15834	10415	14565	0.023
Het-Het	Mean	64942	60036	63979	19034	0.066
*n* = 1069	Median	59996	57677	61836	16364	0.069
	*SD*	12133.74	12516.77	8130.59	13152.80	0.024
Mgeno	*F*	62.185	2.989	25.913	33.116	5.660
df 1, 5830	Sig.	**0.000**	0.084	**0.000**	**0.000**	**0.017**
Ogeno	*F*	6.150	31.956	0.147	7.542	0.166
df 1, 5830	Sig.	**0.013**	**0.000**	0.701	**0.006**	0.683
Sex	*F*	270.866	82.086	68.498	104.964	19.404
df 1, 5830	Sig.	**0.000**	**0.000**	**0.000**	**0.000**	**0.000**
Mgeno * Ogeno	*F*	21.447	6.586	10.616	8.734	15.351
df 1, 5830	Sig.	**0.000**	**0.010**	**0.001**	**0.003**	**0.000**
Mgeno * sex	*F*	0.010	8.003	16.252	13.825	20.085
df 1, 5830	Sig.	0.920	**0.005**	**0.000**	**0.000**	**0.000**
Ogeno * sex	*F*	2.744	14.354	7.391	19.209	0.313
df 1, 5830	Sig.	0.098	**0.000**	**0.007**	**0.000**	0.576

**(A)**							

**Sex**		**Group**	**Start**	**End**	**MF**	**BW**	**Dur**

*F*	Wt-Wt	Het-Wt	0.003	0.134	1.000	0.000	0.000
		Het-Het	0.000	0.570	0.017	0.000	0.004
*M*	Wt-Wt	Het-Wt	0.067	0.217	0.000	1.000	1.000
		Het-Het	0.000	0.000	0.000	0.000	0.164

*Descriptive values and statistics. Mgeno, maternal genotype; Ogeno, offspring genotype; MF, mean frequency; BW, bandwidth; Dur, duration. Main effects (two- and three-way ANOVA) are presented for each model, in the lower part of each table. N, number of calls. Table 2A summarizes the results of post-hoc tests.*

In the Mthfr+/− females, which emit syllables with higher frequencies (start, end, and mean frequency) than males, an interaction between maternal x offspring genotype was observed, where maternal Mthfr+/+ (Wt-Wt) genotype had higher frequencies, compared to pups with maternal Mthfr+/− genotype, and the offspring Mthfr+/− genotype (Het-Het) had lower mean and end frequencies, compared to pups with Mthfr+/+ genotype (Wt-Wt and Het-Wt). In contrast, the start frequency and bandwidth were lower in the mice with maternal Mthfr+/− genotype and further reduced by offspring Mthfr+/− genotype such that the Mthfr+/− pups of Mthfr+/− dams showed the lowest start frequencies. In the males, maternal and offspring Mthfr+/− genotype lowered the frequencies of the start, end, and mean frequency of the syllables. In both sexes, the bandwidth was lowered by maternal Mthfr+/− genotype and further lowered by offspring Mthfr+/− genotype (Table 2). Post-hoc analysis revealed that differences in the start frequency, mean frequency, and bandwidth between the control Wt:Wt group and test groups are found in most comparisons, while an effect of genotype on duration was found only in female pups (Table 2A).

**FIGURE 1:**
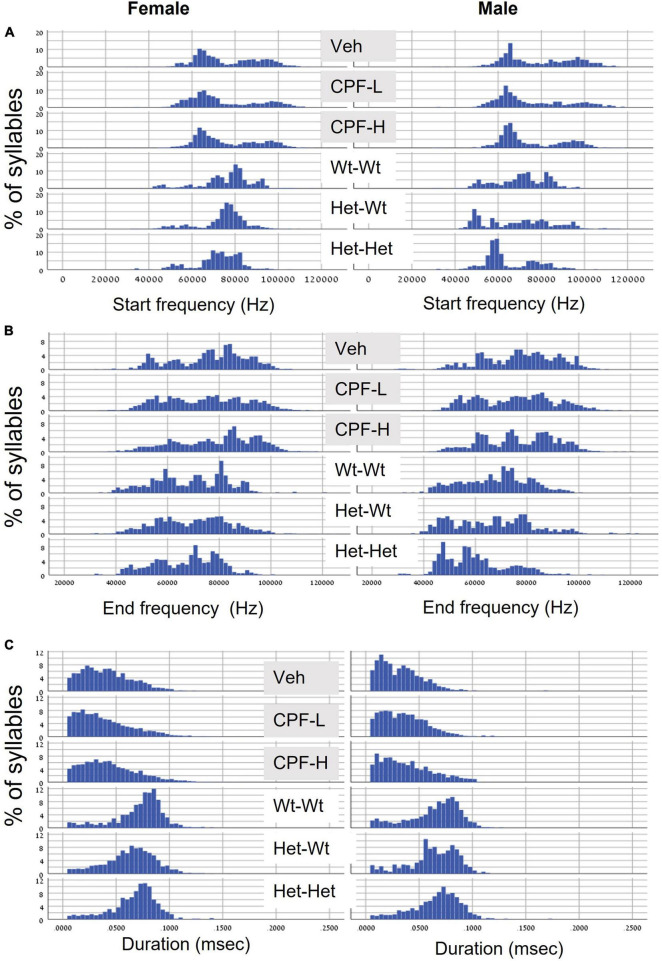
CPF treatment and Mthfr+/– genotype alters the distribution of isolation induced syllables in female and male pups. **(A)** Start frequency. **(B)** End frequency. **(C)** Duration. For each variable, the following groups are presented from top to bottom: Vehicle (Veh), CPF low dose (CPF-L), CPF high dose (CPF-H), Wt:Wt, Het-Wt, and Het-Het.

Next, we examined the distribution of syllable frequencies and range, focusing on the start and end frequencies, and the bandwidth for the effects of sex and the impact of the relevant model variable (CPF treatment or Mthfr+/− genotype). The sex and model differences are illustrated in Figure 1; rows 1–3 CPF model and rows 4–6 Mthfr model. Compared to the males, the female mice were more affected by prenatal CPF for the start frequency, end frequency, and syllable duration (Figures 1A–C, rows 1–3, left side). In the Mthfr+/− males, the distributions of start and end of the syllable were shifted to lower frequencies with shorter duration compared to Wt:Wt (Figures 1A,B, rows 4–6, right side). Comparison and statistical analysis of the effects of treatment and genotype on syllable distribution are shown in Tables 1, 2, respectively.

### Unsupervised Clustering of Isolation Induced Ultrasonic Vocalizations

In order to further analyze the spectral characteristics of the calls, the range of syllables was analyzed and classified on the basis of the frequencies of the different types of calls. This resulted in large variety of syllables that might not have been detected if the syllables were analyzed under a single distribution. This analysis allowed us to detect changes that occurred only in one type of syllable or tendencies to emit a particular type of syllable as a result of the relevant ASD factor (CPF treatment and Mthfr+/− genotype, compared to their controls).

**FIGURE 2:**
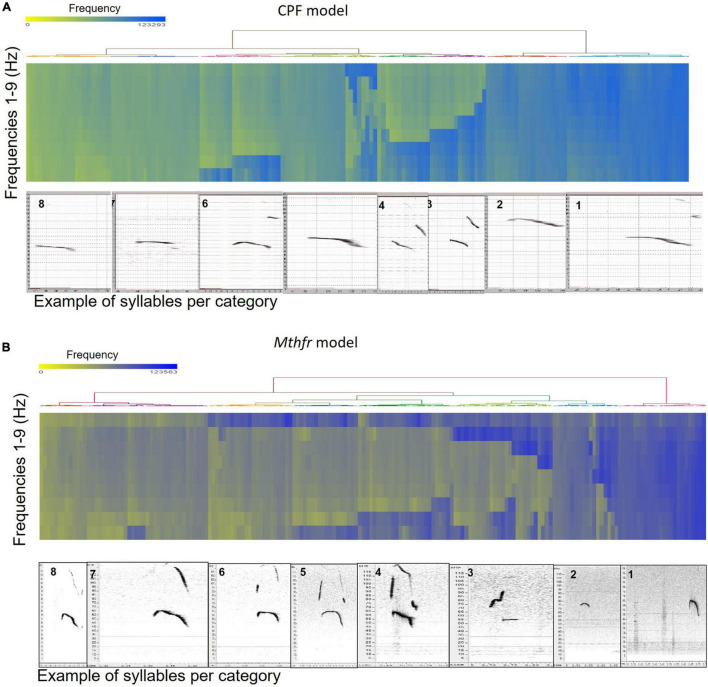
Hierarchical clustering of syllable spectral properties resulted in eight distinct clusters for each ASD model. Hierarchical clustering dendrogram and heat-map for the CPF model **(A)** and the Mthfr model **(B)**. Frequencies in the heat map range from high frequencies in blue to low frequencies in yellow. Examples of syllables from each cluster, as generated by Avisoft SASLab Pro ver5.2.11 software, numbered as in the text and tables, are shown below each heat-map.

**FIGURE 3:**
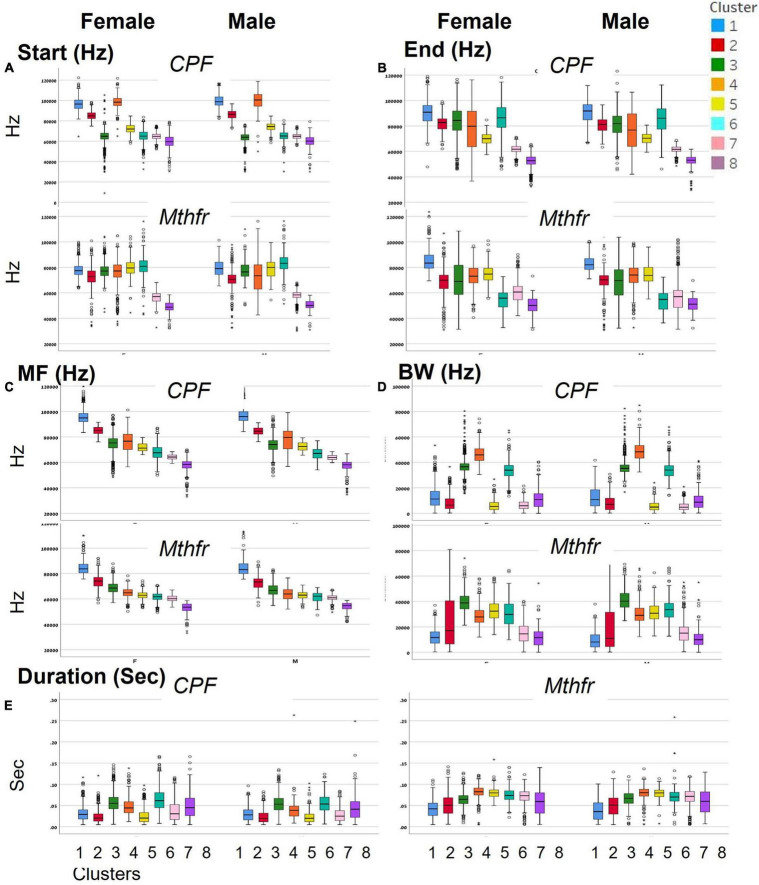
Distinct spectral and temporal characteristics of the syllable clusters. Spectral and temporal characteristics of the syllables in each of the clusters for female and male of CPF and Mthfr models: **(A)** start frequency, **(B)** end frequency, **(C)** mean frequency, **(D)** bandwidth, and **(E)** duration. Median, confidence interval (95 and 5%) and outliers are presented. Note that each ASD model has a unique cluster distribution; the number and color code for Mthfr model do not overlap with the same characteristics in the CPF model.

To categorize all syllables with no *a priori* bias, we used an unsupervised method based on the USV spectral properties, as described above. Each syllable was equally divided into nine frequencies which were submitted to hierarchical clustering. Hierarchical clustering using Euclidean distance as point distance metric and Wards linkage as cluster distance metric resulted in eight distinct clusters in each model. The dendrogram generated by the Partek^®^ software was colored to illustrate the different clusters (Figure 2). The heat-maps indicate the values at 9 points along the syllables’ main frequency from the beginning of the syllable (upper part of the heat-map) to the end of the syllable (lower part of the heat-map), ranging from high frequencies in blue to low frequencies in yellow. Syllables with a single component and narrow bandwidth in a frequency range around 80,000 kHz are shown at the right end of the heat-map, whereas clusters with frequency range around 50,000 kHz and narrow bandwidth are at the left side of the heat-maps. Examples of syllables from each cluster are shown below each heat-map for each of the two ASD models. Syllables containing more than a single component appear at the central region of the heat-maps. The heat-map demonstrates the presence of frequency steps and the relative proportion between the length of each component of the syllables. This presentation highlights differences between the models/strains that are most obvious in heat-maps. For example, the heat map illustrates the higher syllable complexity in the Mthfr model, compared to the CPF model. The differences between the clusters are supported by the differences in start, end, mean frequencies, bandwidth, and duration (see descriptive data in the Supplementary Tables 2A,B). The median values and range of each cluster characteristics are shown in the box plots in Figure 3. Note that although the color code is similar for the CPF and the Mthfr models, these clusters do not represent similar syllables.

In female and male mice of the CPF model, there is a clear difference in the start frequency between clusters 1–3 and 4–6, with clusters 3, 6, 7, and 8 showing lower variability. In the Mthfr model, start frequency of clusters 1–6 are characterized by values around 80,000 kHz while the start frequency of clusters 7 and 8 is around 60,000 kHz. Compared to the start frequency, the end frequency is more variable in both models, with similar patterns in male and female in the two models, as can be seen in Figures 3A,B. In both models, the mean frequency of the clusters declines from cluster 1 to 8, with each cluster having lower mean frequency than the previous. The highest frequency is around 95,000 kHz in the CPF model and 80,000 kHz in the Mthfr model, while the lowest frequencies are 50000–55000 kHz in both models. In contrast to the gradual decline of the mean frequency from cluster 1 to 8, the bandwidth values vary among clusters. In the CPF model, clusters 1, 2 5, 7, and 8 are at the range of 10,000–20,000 kHz, whereas clusters 3, 4, and 6 range around 40,000 kHz. In the Mthfr model, clusters 1, 7, and 8 range below 20,000 kHz, cluster 2 had a large variability, and clusters 3–6 vary between 30,000–40,000 kHz (Figure 3D). Thus, in both models, clusters 3–6 contain syllables with two or three distinct components, as can be observed also in the heat-maps presented in Figure 2. As for the duration (Figure 3E), the clusters in CPF models show a trend similar to that of the bandwidth, where syllables with low bandwidth in the lower frequency range are also shorter and those in the higher frequency range bandwidth are longer. In addition, there are significant sex differences (Supplementary Table 2A). In the Mthfr model, in clusters 1 and 2, syllable duration is 0.051 s or below, whereas in all other clusters syllables are mostly longer than 0.051 s with minimal differences between male and female (Supplementary Table 2B).

### The Effect of Chlorpyrifos and Methylenetetrahydrofolate Reductase on Syllable Properties in Each Cluster

Following characterization of eight distinct syllable clusters in each ASD model, the effects of the independent factors on syllable properties, compared to control, in each of the different clusters were examined. The effect of CPF treatment is shown in Tables 3A,B for female and male mice, respectively. In general, CPF had a greater effect on female pups (Table 3), such that in clusters 1, 3, 6, 7, and 8 at least 4 out of 5 variables were significantly affected. This was more apparent in the CPF-L group (Table 3). The greater effect of the CPF-L group was also apparent in the males with more significant changes in clusters 1, 2, 3, 7, and 8.

**TABLE 3:** The effect of CPF exposure on spectral and temporal properties of isolation induced syllables of the different syllable clusters.

**(A) Female**												
**Cluster**	**T**	**N**	**Start**	**End**	**MF**	**BW**	**Dur**	**Start**	**End**	**MF**	**BW**	**Dur**
		**Unit**	**Hz**	**Hz**	**Hz**	**Hz**	**Sec**	***p***	***p***	***p***	***p***	***p***
1	Veh	309	94745	87800	93473	12875	0.033					
	CPF-L	525	98178	91358	96378	10700	0.026	**0.000**	**0.000**	**0.000**	**0.000**	**0.000**
	CPF-H	503	95577	92207	95844	10995	0.029	0.204	**0.000**	**0.000**	**0.000**	**0.003**
2	Veh	212	84217	82704	85500	6430	0.021					
	CPF-L	276	86188	80675	85139	6448	0.019	**0.000**	**0.001**	0.448	0.168	0.497
	CPF-H	276	84672	83526	86026	6493	0.021	0.338	**0.016**	0.084	0.977	0.798
3	Veh	232	65400	81225	74708	35600	0.058					
	CPF-L	403	64600	83557	75594	36658	0.054	**0.009**	**0.000**	**0.000**	**0.000**	**0.034**
	CPF-H	409	64603	86100	76199	36905	0.054	0.342	**0.000**	**0.004**	**0.000**	**0.025**
4	Veh	74	95803	76369	76984	46193	0.048					
	CPF-L	111	100402	77454	78410	45986	0.040	**0.000**	**0.009**	0.576	0.480	0.148
	CPF-H	192	97741	82684	76682	45600	0.045	**0.021**	**0.002**	0.899	0.231	0.862
5	Veh	97	72106	69300	71300	5400	0.020					
	CPF-L	302	71910	70264	71511	4400	0.018	0.954	0.630	0.759	0.206	0.207
	CPF-H	266	71766	69800	71472	6496	0.024	0.918	0.966	0.553	0.145	0.087
6	Veh	154	65000	86186	68797	32655	0.062					
	CPF-L	299	64882	84866	66799	33150	0.067	**0.009**	**0.047**	**0.000**	0.061	**0.025**
	CPF-H	452	65057	86600	68616	34650	0.058	0.690	0.472	0.371	**0.004**	0.195
7	Veh	144	64508	61414	64910	6228	0.031					
	CPF-L	400	64706	61919	64405	5569	0.026	0.832	**0.044**	**0.005**	0.051	**0.015**
	CPF-H	291	64900	61517	64125	6400	0.036	0.660	0.101	**0.016**	0.902	**0.003**
8	Veh	158	61942	52498	59962	12339	0.039					
	CPF-L	503	58714	53200	57976	8325	0.040	**0.000**	**0.000**	**0.000**	**0.000**	0.264
	CPF-H	225	61200	51700	59676	14370	0.059	0.222	**0.000**	0.787	**0.000**	**0.000**

**(B) Male**												

**Cluster**	**T**	**N**	**Start**	**End**	**MF**	**BW**	**Dur**	**Start**	**End**	**MF**	**BW**	**Dur**

		**Unit**	**Hz**	**Hz**	**Hz**	**Hz**	**Sec**	***p***	***p***	***p***	***p***	***p***
1	Veh	168	98100	91442	95622	10287	0.026					
	CPF-L	378	100795	92086	98547	12452	0.032	**0.000**	**0.039**	**0.000**	**0.023**	**0.007**
	CPF-H	244	97489	91738	94544	7900	0.022	**0.028**	0.177	0.061	**0.008**	0.057
2	Veh	89	85357	80682	84050	5720	0.016					
	CPF-L	261	85990	81500	85111	6142	0.018	0.606	0.579	**0.034**	0.731	0.136
	CPF-H	185	88243	80866	85465	9513	0.025	**0.000**	0.079	**0.043**	**0.005**	**0.000**
3	Veh	94	65100	81110	74815	36100	0.051					
	CPF-L	304	63242	79905	73889	34700	0.052	**0.000**	0.568	**0.008**	**0.000**	0.314
	CPF-H	278	64400	84096	74528	35287	0.057	0.114	**0.003**	0.634	**0.001**	**0.000**
4	Veh	33	99510	74898	80221	47976	0.041					
	CPF-L	52	102203	83521	81621	50148	0.040	0.531	0.425	0.286	0.345	0.900
	CPF-H	50	99300	74355	79471	46796	0.037	0.253	0.618	0.539	0.632	0.849
5	Veh	54	73601	67300	71433	5825	0.018					
	CPF-L	190	74444	70289	73020	4524	0.021	0.168	**0.002**	**0.018**	0.105	0.521
	CPF-H	109	74716	71110	73305	4588	0.018	0.468	**0.005**	0.262	0.114	0.148
6	Veh	29	64200	84400	65144	33900	0.046					
	CPF-L	156	64707	86849	67446	33154	0.051	0.670	0.426	0.103	0.437	0.167
	CPF-H	193	65889	85900	67567	34200	0.058	0.087	0.437	0.133	0.697	**0.006**
7	Veh	57	65109	62023	64500	4400	0.020					
	CPF-L	303	64636	60753	63678	5388	0.027	0.085	**0.000**	**0.011**	0.090	**0.025**
	CPF-H	233	64829	62321	64305	3950	0.025	0.224	0.618	0.644	0.508	0.217
8	Veh	51	60180	51857	57576	11990	0.032					
	CPF-L	281	59881	53468	58595	8126	0.044	0.346	**0.000**	**0.006**	**0.004**	**0.002**
	CPF-H	111	60700	52888	59347	11207	0.044	0.289	0.218	**0.020**	0.630	**0.021**

*Median values for the CPF mice: A, female; B. male. The five columns on the right show the *p* values of the Kolmogorov-Smirnov test for the effect CPF. Bold values indicate a significant effect compared to the vehicle group. CPF-L, CPF low dose; CPF-H, CPF high dose; MF, mean frequency; BW, bandwidth; Dur, duration; T, treatment; N, number of calls.*

Since in the Mthfr model most of the significant effects were observed in males, we present the data for males only (Table 4). Maternal and offspring genotype affected syllable variables as follows: an effect of maternal and offspring Mthfr+/− genotypes was noticed in clusters 2, 3, 6, 7, and 8 with higher number of differences detected due to offspring genotype (Wt:Wt vs. Het:Het). In addition, the variables start frequency and bandwidth were affected in a higher number of clusters compared to the rest of the variables.

**TABLE 4:** The effect of Mthfr genotype on spectral and temporal properties of isolation induced syllables of the different syllable clusters in male pups.

**Cluster**	**group**	**N**	**Start**	**End**	**MF**	**BW**	**Dur**	**Start**	**End**	**MF**	**BW**	**Dur**
			**Hz**	**Hz**	**Hz**	**Hz**	**Sec**	***p***	***p***	***p***	***p***	***p***
1	Wt-Wt	197	77750	82194	82569	8893	0.037					
	Het-Wt	51	81155	82514	83709	5841	0.033	**0.000**	0.167	**0.002**	0.214	0.410
	Het-Het	81	78144	81780	83408	8280	0.038	0.092	0.352	0.199	0.997	0.375
2	Wt-Wt	213	70209	71362	73198	9029	0.048					
	Het-Wt	43	70433	68532	71942	9918	0.041	0.080	**0.003**	0.479	0.921	0.167
	Het-Het	72	73763	67228	74592	20463	0.056	**0.000**	**0.000**	**0.016**	**0.001**	0.067
3	Wt-Wt	226	74706	64896	64820	40230	0.066					
	Het-Wt	56	76642	71309	64357	39205	0.063	0.081	**0.042**	0.876	0.802	0.952
	Het-Het	98	77555	74903	68210	41504	0.069	**0.003**	**0.000**	**0.000**	0.549	0.276
4	Wt-Wt	261	73237	73605	62925	28851	0.081					
	Het-Wt	83	69884	76161	65278	30904	0.078	**0.030**	0.215	0.057	**0.000**	0.070
	Het-Het	37	77042	74185	64447	27104	0.086	**0.008**	0.106	0.065	**0.001**	0.121
5	Wt-Wt	116	81653	72528	63783	33386	0.080					
	Het-Wt	69	79944	73142	61075	32396	0.078	**0.001**	0.483	0.378	**0.032**	0.913
	Het-Het	52	77061	74454	63378	28061	0.080	0.286	**0.000**	0.375	**0.003**	0.085
6	Wt-Wt	208	83676	51807	62154	34805	0.072					
	Het-Wt	57	80736	51540	58952	33697	0.068	**0.000**	0.605	0.519	**0.000**	0.622
	Het-Het	59	83566	56569	62890	29683	0.070	**0.039**	**0.006**	0.759	0.114	**0.000**
7	Wt-Wt	182	58469	64256	61461	18256	0.079					
	Het-Wt	95	52209	61334	60223	16457	0.062	**0.001**	0.194	**0.000**	**0.000**	**0.018**
	Het-Het	563	58542	54548	60916	13815	0.072	**0.000**	**0.000**	**0.003**	**0.000**	**0.000**
8	Wt-Wt	86	51791	51538	56059	9456	0.073					
	Het-Wt	86	49137	49198	53585	11866	0.067	**0.000**	**0.040**	0.317	**0.000**	0.157
	Het-Het	107	50758	51892	55036	7447	0.039	**0.047**	**0.001**	**0.000**	**0.011**	0.319

*Median values are presented. The five columns on the right show the p values of the Kolmogorov-Smirnov test for the effect of Mthfr genotype. Bold values indicate a significant effect compared to the Wt:Wt group. MF, mean frequency; BW. bandwidth; Dur, duration; T, treatment; N, number of calls.*

Thus, in both models, the start and end frequencies and the bandwidth were related to the ASD factor.

When evaluating in both models the effects of the independent factors on syllable variables in each cluster, two qualitative features are salient: (1) the start frequency and bandwidth were the variables that were affected by the ASD factor in the greatest number of clusters and (2) bandwidth and duration were the variables that show the highest percent change compared to controls group (Tables 3, 4).

### The Effect of Chlorpyrifos and Methylenetetrahydrofolate Reductase on Syllable Usage

**FIGURE 4:**
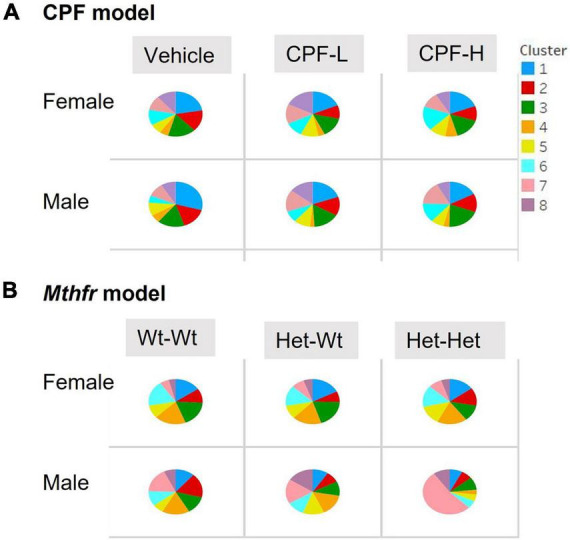
CPF and Mthfr+/– altered the proportion of syllables per cluster emitted by the pups. The proportion of syllables emitted by each experimental group is presented for females and males: **(A)** CPF model and **(B)** Mthfr model. Different clusters are color coded as in Figure 4. Note that each ASD model has a unique cluster distribution; the number and color code for Mthfr model do not overlap with the same characteristics in the CPF model.

Last, we examined the proportional production of the different syllables, categorized by cluster number. The percent of syllables from each cluster emitted by the pups of the different groups is shown in Figure 4. In both models, a chi square test for the distribution of the percent of calls from each cluster revealed significant differences compared to the model’s control group (Veh and Wt:Wt). In the CPF model, compared to the vehicle group, mice of both sexes that were exposed to both doses of CPF emitted a lower proportion of high frequency syllables (clusters 1, 2, 3). Mice that were exposed to the low dose (CPF-L) showed a higher proportion of low frequency cluster 5 syllables. Both doses of CPF also increased the proportion of low frequency clusters 7 in males. In the females, only the low dose CPF-L mice showed a higher proportion of clusters 7 and 8. CPF-H females had a 30–50% higher proportion of low frequency and two components syllables (clusters 4, 5, and 6) (Figure 4A). Interestingly, the male offspring of maternal Mthfr+/− genotype (Het-Wt and even more in the Het-Het pups) showed a similar tendency, such that there was a lower proportion of syllables with high frequency in syllables of both simple (Clusters 1 and 2) or complex (3, 4, 5, and 6) structure (20–75% of control). Maternal Mthfr+/− genotype also was associated with a marked increase in the proportions of low frequency calls (clusters 7 and 8). Moreover, an additional effect of offspring genotype was shown in cluster 7, where male Het:Het mice showed a 400% increase in the proportion of those syllables (Figure 4B). Altogether, CPF and Mthfr genotype decreased the proportion of syllables at the range of 70,000 kHz and above and increased the use of syllables with a range around 50,000 kHz.

## Discussion

Comparative exploration of the spectral and temporal aspects of isolation USVs in pups of the two ASD models shows that: (1) the start frequency, end frequency, and bandwidth were the variables most significantly affected by the ASD factor in most clusters and (2) bandwidth and duration showed the largest differences between ASD and control groups. These findings were distinct for syllables with a simple structure, i.e., a single main component. By analyzing syllable characteristics for two distinct mouse models, we found similar communication deficits, despite the fact that the two models were derived from different strains that had discrete basal USV characteristics. We suggest that these variables are the most sensitive to the developmental changes in brain circuitry in ASD models.

In addition to the number of syllables, syllable duration is the most frequently used measure to characterize isolation induced calls in pups as summarized by Young et al. (2010), and Lai et al. (2014). Shorter syllable duration compared to their controls was previously reported in the C57-htr1a; C57-cacna1c; FVB MALTT, and C57-Ext1c; ProSap1/Shank 2 (Ey et al., 2013) strains, whereas C57-Tsc2 het and male FMR1-KO pups (Nolan et al., 2020) had longer syllable durations. Most of these studies do not distinguish between syllable types. Mice exposed to CPF had shorter syllable durations regardless of syllable type or sex similar to FMR1-KO mice (Lai et al., 2014). This was more pronounced in the females’ simple syllables in the cluster analysis. A similar observation was obtained in the Mthfr model, where syllable cluster analysis enabled detection of shorter duration in simple syllables with low frequency (clusters 6–8) of the experimental groups vs. the control. Improvement in the resolution and specificity of the effect to a particular syllable type was reported for the BTBR mouse strain (Scattoni et al., 2008b), where the duration of harmonic and composite, but not other syllables, differs compared to other strains. Thus, when the difference between the control and ASD model mouse occurs in the predominant syllable type, changes in the spectral and temporal properties of all syllables can be detected.

Various classification schemes of syllable types and characteristic specifications were suggested by Scattoni et al. (2008a, b), Young et al. (2010), and Ey et al. (2013), enhancing the ability to detect specific alterations in model pups, and detection of inter-strain differences. Classification of multiple (10–100) syllable types performed by the observer or software designed for that task (Van Segbroeck et al., 2017; Coffey et al., 2019) provides the desired enhanced resolution. Alternative strategies of analysis rely on the use of fewer and more general categories of calls as reported by several groups in recent years (Agarwalla et al., 2020; Nolan et al., 2020; Yang et al., 2021). Aiming to exploit syllable classification to homogeneous categories while at the same time avoiding over-categorization, we implemented an unsupervised method of hierarchical clustering based on the spectral properties of the syllables. The number of clusters was restricted to 8, as a compromise between overfitting of clusters to syllables and the need to have a comprehensive portrayal of the range of clusters. Although only spectral information was used for classification, the clusters were distinct in their temporal properties (i.e., duration) as evident in Figure 3E and Tables 3, 4 confirming the relevance of the cluster analysis to create meaningful categories of syllables. The unbiased classification enabled the detection of the effect of CPF treatment on the mean frequency, and syllable bandwidth, that were not detected before in the CPF model (Berg et al., 2020; Morales-Navas et al., 2020).

Although we did not find any deficits in the latency to pup retrieval in either the CPF or Mthfr model at a young age, a more careful analysis of the dams’ response to recorded USVs, without the olfactory cues that the “lost” pup provides, will shed light on the functional significance of the “ASD-like” spectro-temporal properties. In the TBX1 genetic model, dams responded more to the location of signals that had sequence properties of WT mice, suggesting that the higher order characteristics of the call sequence affect maternal response (Kato et al., 2021).

Another aspect shown to affect quantity and properties of isolation calls is related to anxiety as shown in early works (Olivier et al., 1998) that tested the effect of anxiolytic drugs on USV emission. Benzodiazepines significantly affect the number of calls emitted (Hodgson et al., 2008; Takahashi et al., 2009), suggesting the involvement of the GABAergic system in the regulation of this behavior. Changes in cellular organization of Parvalbumin interneurons and decreased levels of GABAergic proteins were reported in the Mthfr deficient mouse (Sadigurschi and Golan, 2018; Orenbuch et al., 2019) and may influence isolation calls emitted by Mthfr deficient pups. Interestingly, enhanced anxiety was observed in adult offspring to Mthfr deficiency dams (Sadigurschi and Golan, 2018). GABA has also been implicated in the effects of exposure to CPF on anxiety, while perinatal CPF increased anxiety in female mice, but reduced anxiety in zebrafish larvae (Braquenier et al., 2010; Venerosi et al., 2010; Richendrfer et al., 2012; Perez-Fernandez et al., 2020), although the underlying mechanism is unknown. The GABAergic pathway and parvalbumin interneurons undergo dramatic developmental maturation at the first postnatal week (Rivera et al., 1999; Huang et al., 2007) that may affect USV development. Support for the role of GABA in the development of USVs is provided by the finding that premature hypothalamic Agrp-GABAergic neurons, prior to the maturation of the GABA pathway, activate the neuronal circuitry regulating the emission of isolation induced USVs. These neurons are not only involved in the stimulation of USV emission but also affect the repertoire of syllables emitted by the pups (Zimmer et al., 2019). This emphasizes the relation between the maturation of the GABAergic system and aspects of pup-caregiver bonding, supporting the modulation of GABA pathway as a shared origin for the communication deficit in ASD mice models. Changes in the GABA pathway were shown in several mouse models of ASD, including BTBR strain (Han et al., 2012, 2014; Silverman et al., 2015), the FMR1 (Silverman et al., 2015), SCN1A (Han et al., 2012), and others. Although the GABA pathway was less well studied in rodents following prenatal CPF exposure, two recent publications support this direction; mice exposure to CPF, gestation up to the age of weaning, led to decreased expression of the *Gabbr2* (Pallotta et al., 2017) and to increased extracellular GABA concentration in the cerebellum of male, but not female rats (Gómez-Giménez et al., 2018). Further studies are required to link these changes with the modulation of isolation calls emitted by pups in murine models of ASD.

Last, the effect of sex on the number of isolation calls and their characteristics varies between studies. Although early reports do not find an effect of sex (Scattoni et al., 2008b; Lai et al., 2014) the possibility of sex specific effects in the context of ASD promoted analysis of this variable. FMR1-KO (Reynolds et al., 2016; Nolan et al., 2020), PTEN-KO (Binder and Lugo, 2017), and FoxP1-KO mice (Fröhlich et al., 2017) present sex-specific effects on the number and properties of isolation-induced calls. We found a sex difference in the spectral and temporal properties of the calls in both control groups indicating a sex difference in each of the background strains. The interaction between each ASD factor with sex parallels similar complex interactions with sex on social variables in the CPF and Mthfr models (Sadigurschi and Golan, 2018; Lan et al., 2019).

One limitation of the current study is the use of different background strains for the different ASD models. While on one hand, the method applied for syllable classification enabled the detection of similar trends in the two models despite of the strain difference, generalization of the findings to other ASD models remains to be done in future studies.

In summary, genetic and environmental ASD factors had parallel effects on the development of USVs. Further research is needed to explore the association between USV and core behavioral deficits in mouse ASD models.

## Data Availability Statement

The original contributions presented in the study are included in the article/Supplementary Material, further inquiries can be directed to the corresponding authors.

## Ethics Statement

The animal study was reviewed and approved by Israel Council on Animal Care and approved by the Animal Care and Use Committee of Ben-Gurion University of the Negev, Israel.

## Author Contributions

IS performed experiments, developed method for analysis, and analyzed the data. SG, ER, and MW performed experiments. VC-C guided and supervised analysis methods and manuscript critical review. DL performed methods development and manuscript review. OK and HG conceptualized and planned the experiments, supervised experiments and analysis, funded the study, and wrote the manuscript. All authors contributed to the article and approved the submitted version.

## Conflict of Interest

The authors declare that the research was conducted in the absence of any commercial or financial relationships that could be construed as a potential conflict of interest.

## Publisher’s Note

All claims expressed in this article are solely those of the authors and do not necessarily represent those of their affiliated organizations, or those of the publisher, the editors and the reviewers. Any product that may be evaluated in this article, or claim that may be made by its manufacturer, is not guaranteed or endorsed by the publisher.
