# Supplementary material for: Isolation-Induced Ultrasonic Vocalization in Environmental and Genetic Mice Models of Autism
Source: Front Neurosci. 2021 Nov 22;15:769670. doi: 10.3389/fnins.2021.769670 (PMC8645772; doi:10.3389/fnins.2021.769670)
Supplement: Supplementary file 1 [file Presentation_1.PPTX]

## Slide 1
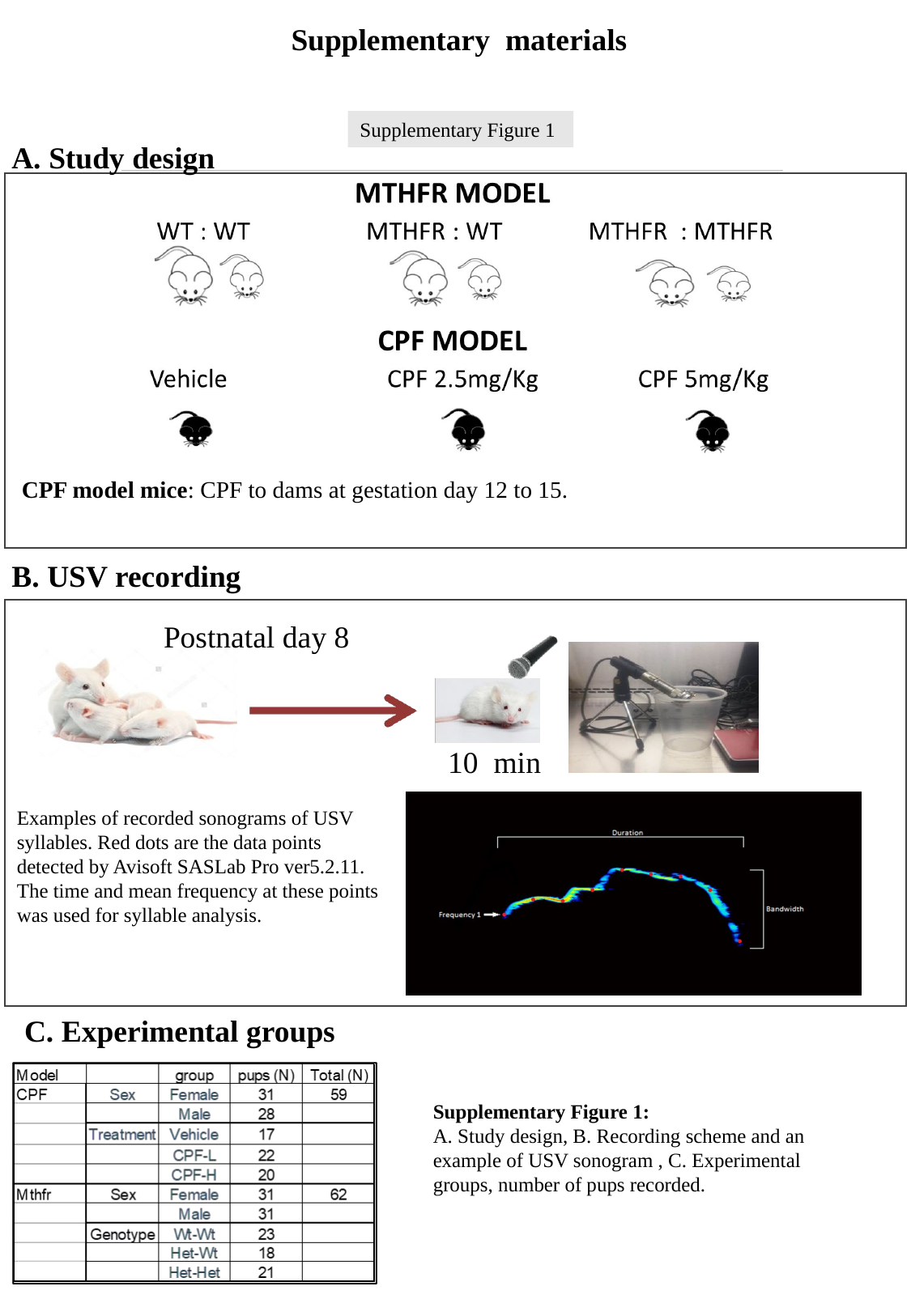

Supplementary materials
Supplementary Figure 1
A. Study design
CPF model mice: CPF to dams at gestation day 12 to 15.
B. USV recording
Postnatal day 8
10 min
Examples of recorded sonograms of USV syllables. Red dots are the data points detected by Avisoft SASLab Pro ver5.2.11. The time and mean frequency at these points was used for syllable analysis.
C. Experimental groups
Supplementary Figure 1:
A. Study design, B. Recording scheme and an example of USV sonogram , C. Experimental groups, number of pups recorded.

## Slide 2
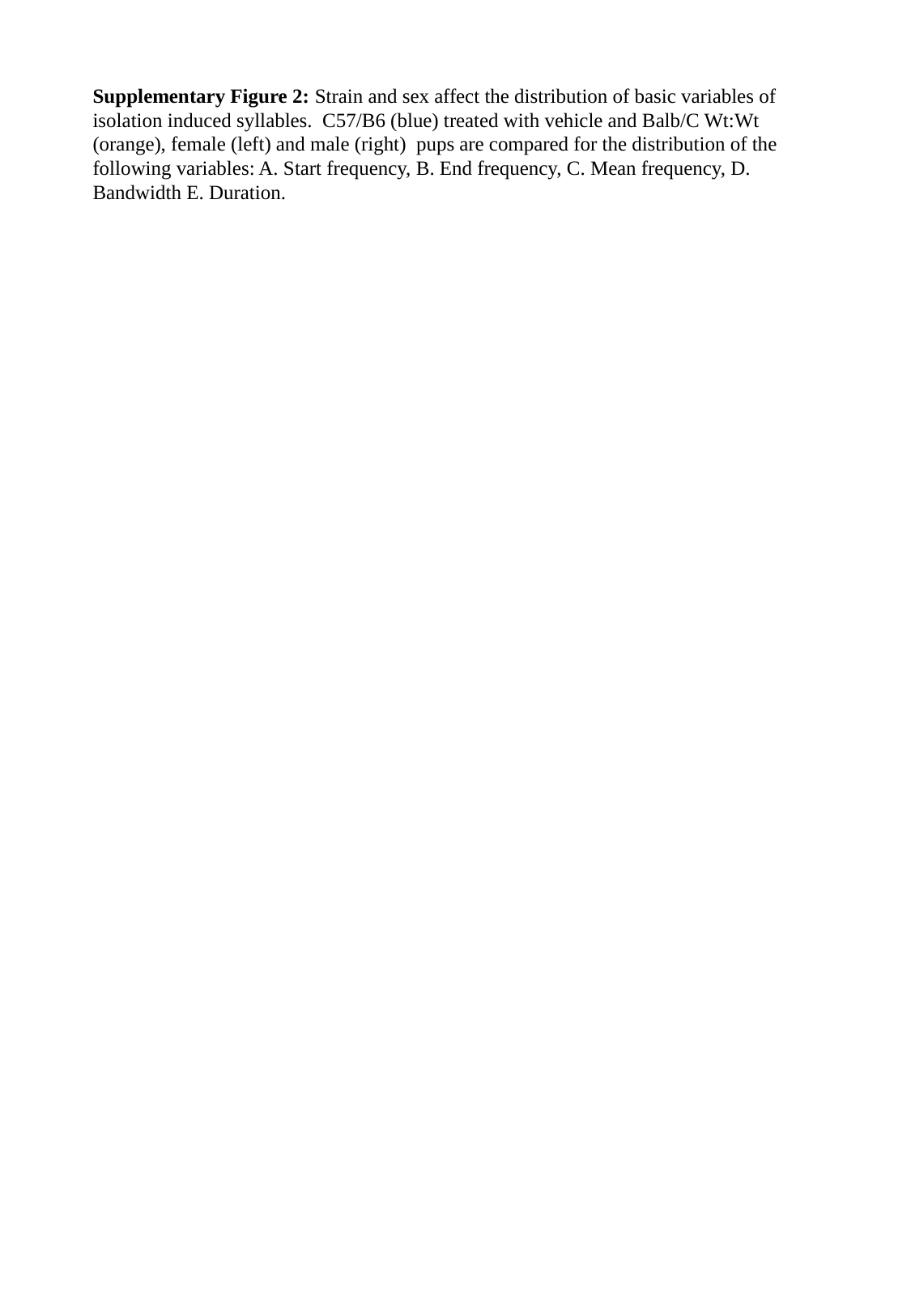

Supplementary Figure 2: Strain and sex affect the distribution of basic variables of isolation induced syllables. C57/B6 (blue) treated with vehicle and Balb/C Wt:Wt (orange), female (left) and male (right) pups are compared for the distribution of the following variables: A. Start frequency, B. End frequency, C. Mean frequency, D. Bandwidth E. Duration.

## Slide 3
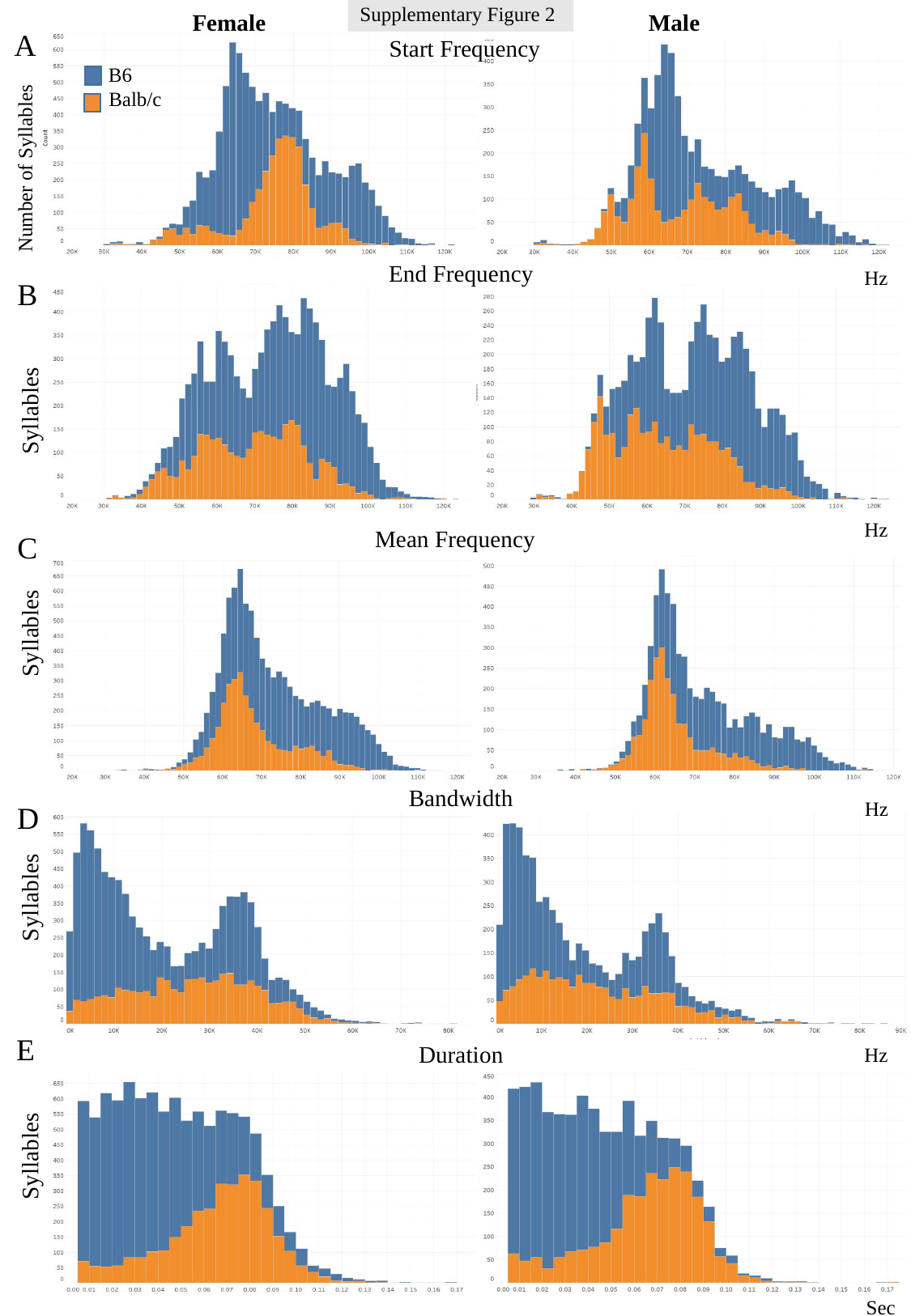

Supplementary Figure 2
Female
Male
Start Frequency
Number of Syllables
End Frequency
Syllables
Mean Frequency
Syllables
Bandwidth
Syllables
Duration
Syllables
B6
Balb/c
A
Hz
B
Hz
C
Hz
D
E
Hz
Sec

## Slide 4
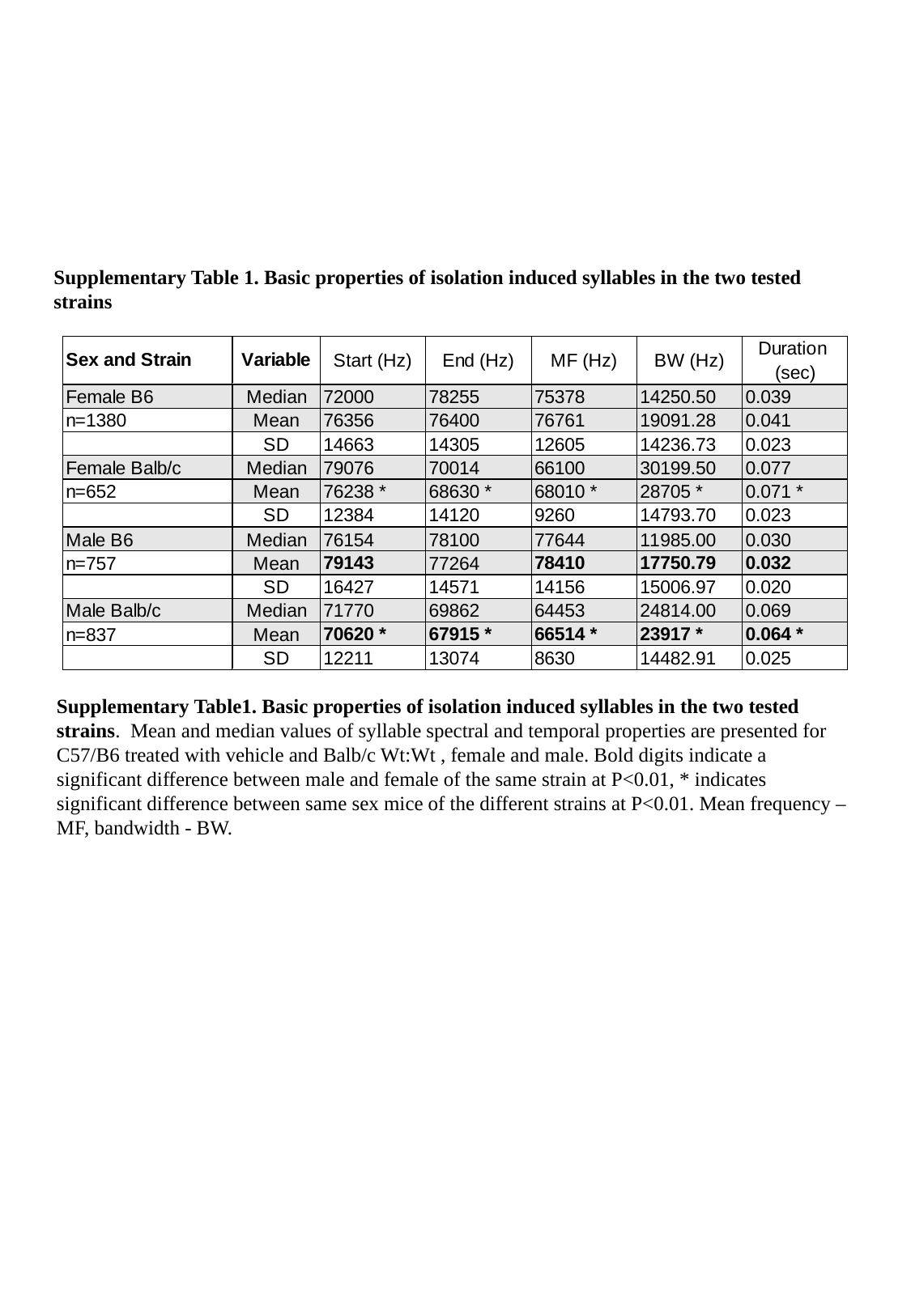

Supplementary Table 1. Basic properties of isolation induced syllables in the two tested strains
Supplementary Table1. Basic properties of isolation induced syllables in the two tested strains. Mean and median values of syllable spectral and temporal properties are presented for C57/B6 treated with vehicle and Balb/c Wt:Wt , female and male. Bold digits indicate a significant difference between male and female of the same strain at P<0.01, * indicates significant difference between same sex mice of the different strains at P<0.01. Mean frequency – MF, bandwidth - BW.

## Slide 5
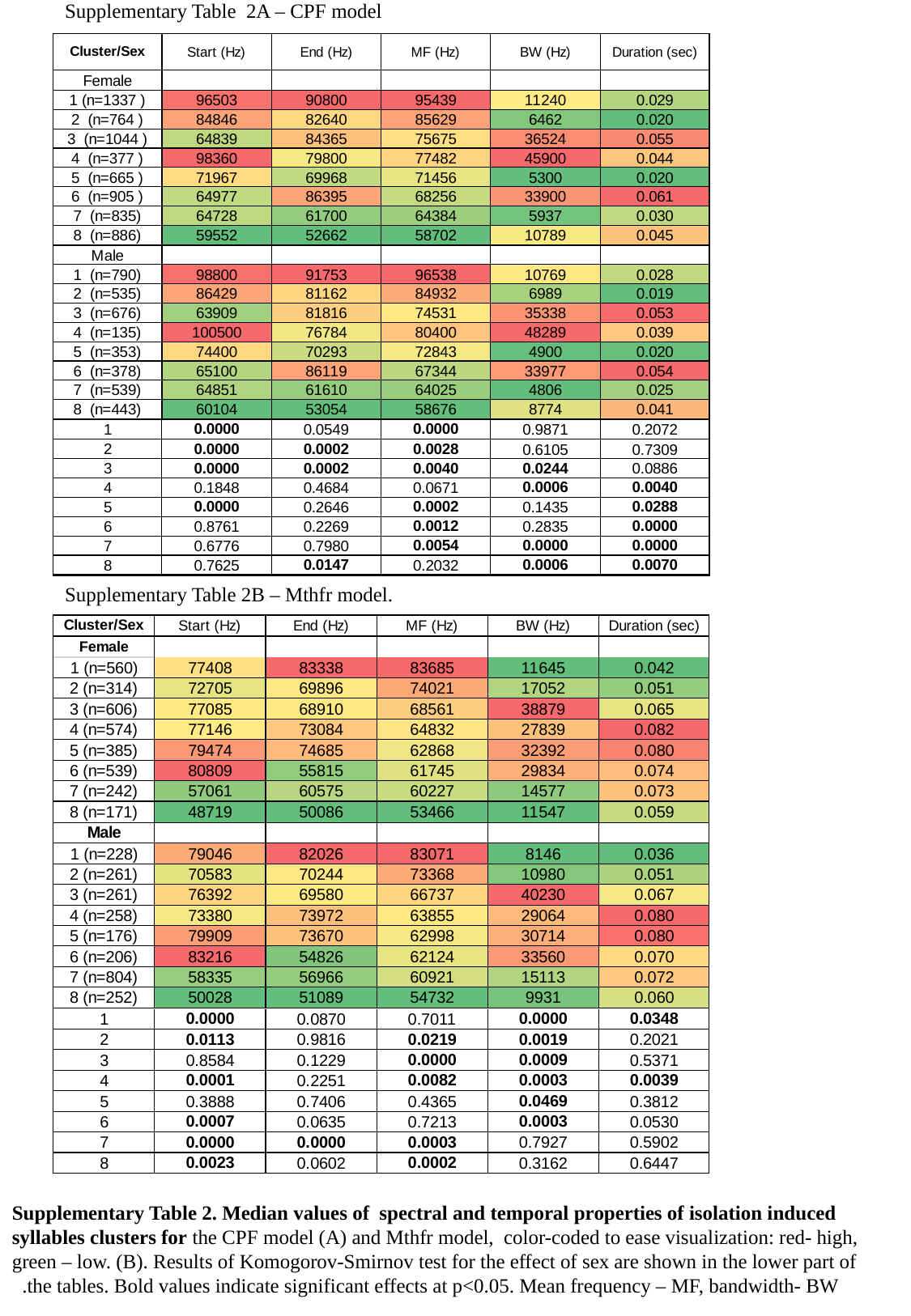

Supplementary Table 2A – CPF model
Supplementary Table 2B – Mthfr model.
Supplementary Table 2. Median values of spectral and temporal properties of isolation induced syllables clusters for the CPF model (A) and Mthfr model, color-coded to ease visualization: red- high, green – low. (B). Results of Komogorov-Smirnov test for the effect of sex are shown in the lower part of the tables. Bold values indicate significant effects at p<0.05. Mean frequency – MF, bandwidth- BW.
